# Navigating complex arterial reconstruction in living donor liver transplantation: the role of the splenic artery as a viable conduit

**DOI:** 10.1186/s12893-024-02709-4

**Published:** 2025-01-06

**Authors:** Hosam Hamed, Mohamed Elshobary, Tarek Salah, Ahmad M. Sultan, El-sayed Abou El-Magd, Ahmed M. Elsabbagh, Ahmed Shehta, Mohamed Abdulrazek, Waleed Elsarraf, Mohamed A. Elmorshedi, Mostafa Abdelkhalek, Usama Shiha, Hassan Magdy Abd El Razek, Mohamed Abdel Wahab

**Affiliations:** 1 Liver Transplant Unit, Gastrointestinal Surgical Center (GISC), Surgery Department, Mansoura University, Mansoura, Dakahleyya, Egypt; 2 Liver Transplant Unit, Gastrointestinal Surgical Center (GISC), Anesthesia Department, Mansoura University, Mansoura, Dakahleyya, Egypt; 3 Liver Transplant Unit, Gastrointestinal Surgical Center (GISC), Radiology Department, Mansoura University, Mansoura, Dakahleyya, Egypt

**Keywords:** Living donor liver transplantation, Hepatic artery, Splenic artery, Reconstruction

## Abstract

**Background:**

In living donor liver transplantation (LDLT), maintaining hepatic arterial flow is critical for graft survival. Alternative methods are required when the recipient’s hepatic artery is unsuitable due to extensive dissection or inadequate flow. This study evaluates the efficacy and safety of splenic artery transposition (SAT) for hepatic arterial reconstruction in LDLT.

**Methods:**

This retrospective cohort study included 10 LDLT patients with hepatic arterial reconstruction by SAT to assess operative parameters, postoperative complications, mortality, and patency rate.

**Results:**

The splenic artery was used because of arterial dissection (70%) or inadequate arterial blood flow. Operative time ranged from 640 to 1020 min, and no splenic infarction was observed. Post-operative complications were as follows; biliary leakage (10%), pancreatitis (10%), intraabdominal hemorrhage (10%), and arterial thrombosis (10%). Mortality in this cohort was 30%, one of them was due to thrombosis of the conduit and the other two died from sepsis-related complications. With a median follow-up of 43 months, this technique was associated with a 70% survival rate.

**Conclusion:**

The splenic artery is a viable conduit for hepatic arterial reconstruction in LDLT, demonstrating an acceptable safety profile and complication rates. This approach is recommended in cases where the recipient’s hepatic artery is significantly compromised.

## Introduction

Hepatic arterial reconstruction is a demanding step in living donor liver transplantation (LDLT). LDLT presents unique challenges for arterial reconstruction compared to deceased donor transplantation. These challenges include the smaller diameter of the recipient’s arteries and a limited range of reconstruction techniques [1, 2]. Good hepatic arterial flow is mandatory for graft function after LDLT [3]. Hepatic artery thrombosis (HAT) carries a 30–50% risk of secondary graft failure with a mortality risk of 50% [4, 5]. In the long term, impaired arterial flow mainly contributes to ischemic biliary strictures [6].

Anatomical arterial reconstruction between the graft and the recipients’ left or right hepatic artery is the standard practice in most centers [7], as more than 90% of arterial reconstructions are anatomical ones [8]. Many LDLT centers utilize the left hepatic artery for arterial reconstruction, to avoid compromising the vascularity of the biliary system from the right hepatic artery [9, 10]. However, anatomical reconstruction may not be feasible in some instances due to inadvertent dissection, luminal discrepancy, previous intra-arterial locoregional therapy, previous surgical scarring, or inadequate length [8, 11]. Substitutes for hepatic artery in extra-anatomical reconstruction include the right gastro-epiploic artery [12], left gastric artery [13], middle colic artery [14], splenic artery [3], and various interposition grafts [15, 16].

Few studies have exclusively studied the feasibility and outcome of extra-anatomical arterial reconstruction through the splenic artery [3]. This study aims to demonstrate the institutional experience of 17 years of right lobe LDLT with special emphasis on SAT. By doing so, we seek to provide valuable insights into this technique’s feasibility, challenges, and outcomes, contributing to the optimization of LDLT practices.

## Patients and methods

### Study design and participants

This is a retrospective cohort investigation of all patients who underwent SAT for hepatic artery reconstruction during the LDLT procedure performed at the Liver Transplantation Unit, Gastrointestinal Surgical Center, Mansoura University, Egypt. The study included the patients who were operated on during the period between May 2004 and January 2021. The included donors aged between 21 and 45 years and were first-to-fourth-degree relatives of the recipient.

### Ethical considerations

The transplantation process was individually revised and approved by the local ethical committee of our medical school, along with the National Committee for Organ Transplantation. The informed consent process involves a comprehensive and detailed discussion between the surgeon, donors, recipients, and their families. Specific risks and complications, including but not limited to bleeding, leakage, infection, thrombosis, and graft dysfunction, are explicitly outlined. Additionally, we emphasize the potential need for re-intervention or additional surgical procedures if complications arise. Informed consent was obtained from the donor before the evaluation process and another before the surgery.

### Preoperative evaluation

During the multi-step planned process of donor evaluation [17], his/her hepatic arterial system was visualized via computed tomography (CT) angiography, along with the portal and hepatic venous systems. In addition, residual liver volume (RLV) was assessed via CT volumetry, and the graft-recipient weight ratio (GRWR) was calculated. The subject was enrolled as an accepted donor when the RLV ≥ 30% and GRWR > 0.8. On the recipient side, they were all evaluated via triphasic pelviabdominal CT with the reconstruction of the portal venous, hepatic venous, and arterial systems. Reconstruction of the arterial system defines the anatomy and state of the arteries. The hepatic arterial angiography is not routinely performed for the recipients in our center.

### Surgical procedure

The LDLT operative technique has been previously described [18–20]. Our standard approach was to use the recipient’s right or left hepatic artery for the arterial reconstruction of the graft. However, when mild to moderate intimal dissection was encountered, we initially tried to trim the arterial edges until reaching a healthy part. In severe cases, four-quadrant intimal fixation was performed via inside-to-outside prolene 8/0 sutures, keeping the knot outside the vascular lumen. This was followed by an arterial anastomosis in an end-to-end fashion using inside-to-outside double-needled sutures of the same material.

**Fig. 1:**
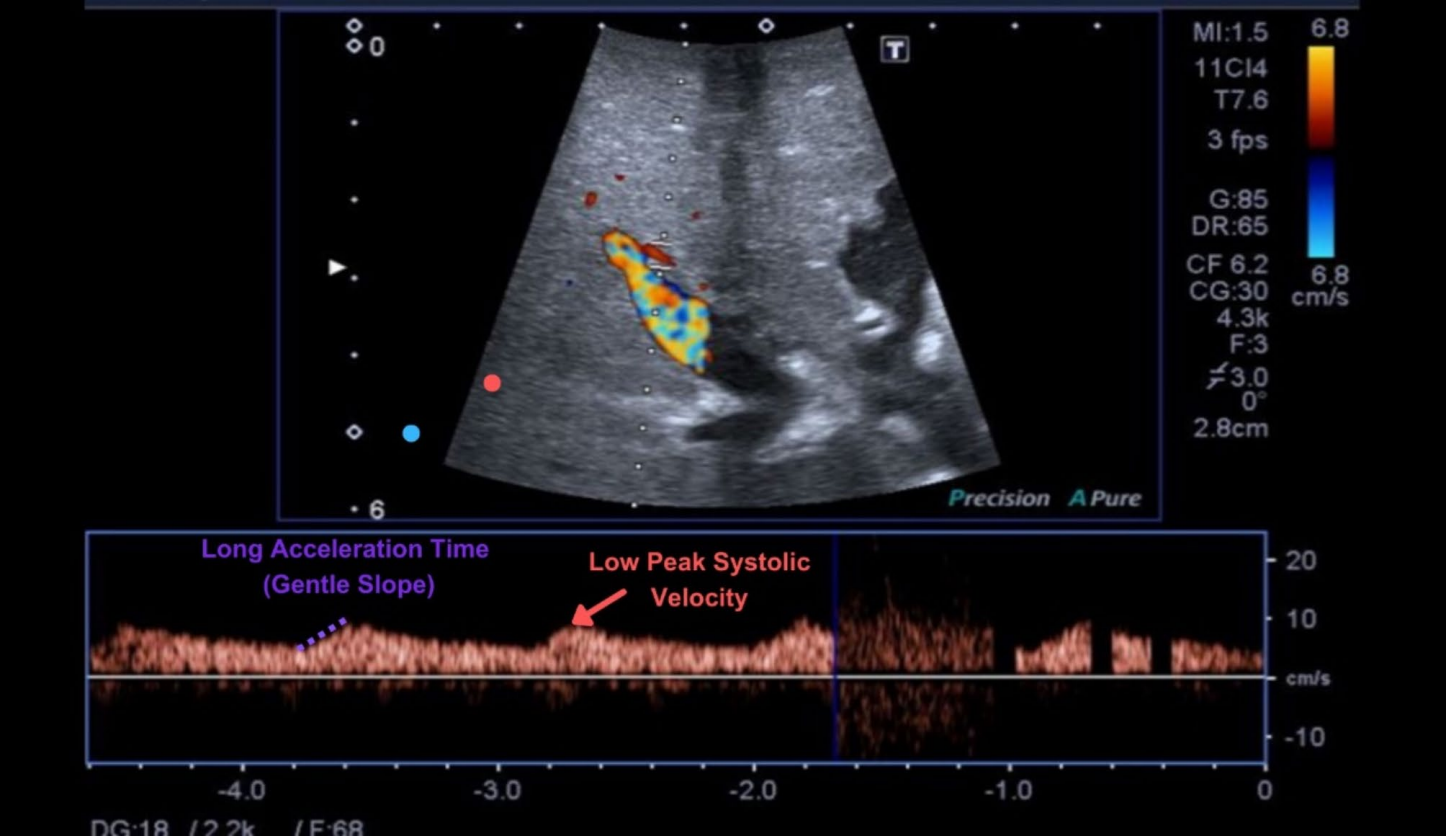
Intraoperative color Doppler image showing impaired hepatic artery flow after reconstruction, characterized by prolonged acceleration time, absence of high peak systolic velocity, and a low resistive index

**Fig. 2:**
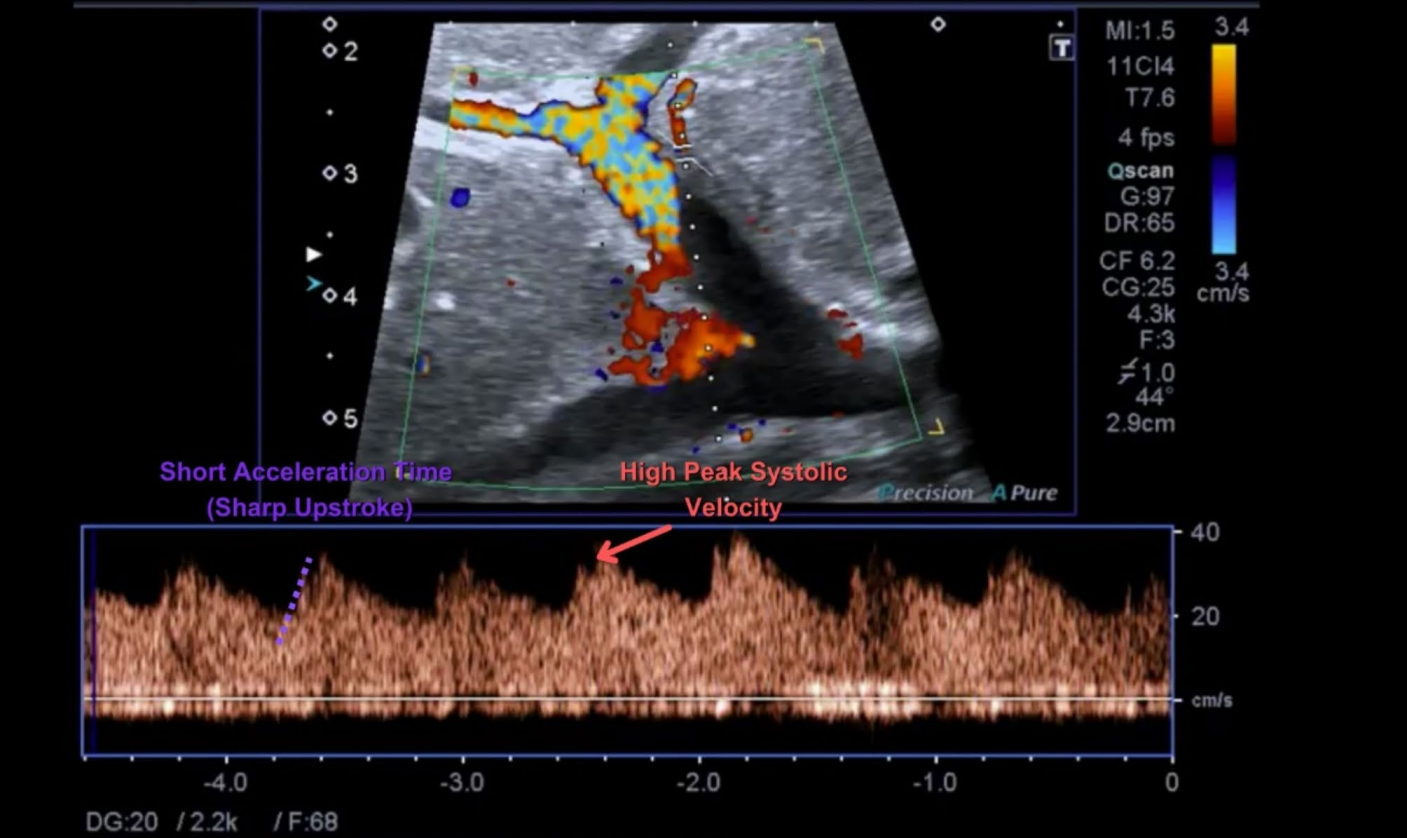
An intraoperative color Doppler image of the hepatic artery following SAT, demonstrating a restored sharp systolic upstroke, reduced acceleration time, and increased peak systolic velocity. High diastolic flow is typically observed immediately after hepatic artery reconstruction, attributed to post-reperfusion hyperemia resulting from ischemia

An intraoperative color Doppler ultrasound was performed to monitor blood flow in the hepatic artery, Figs. 1 and 2. Measurements such as peak systolic velocity, resistive index, and acceleration time were used to assess blood flow quality. If these measurements indicated compromised flow, such as a decrease in peak systolic velocity or a change in waveform pattern, a conservative approach involving watchful waiting and local anesthetic administration was initially employed. However, if the flow impairment persisted or worsened, surgical intervention was necessary to restore adequate blood flow to the transplanted liver.

For severely damaged arteries, not allowing the previous modifications or inadequate flow post-traditional reconstruction, SAT was done. A segment of the splenic artery, about four or five cm long, was obtained by distal ligation of the splenic artery near the spleen. This was followed by dividing three or four pancreatic branches with complete preservation of the splenic vein. The splenic artery was controlled proximally by a bulldog clamp, divided just proximal to the ligation point, and then turned to the other side for arterial anastomosis. The anastomosis was done between the divided end of the splenic artery, supplied by the celiac axis, and the arterial system of the implanted graft. The anastomosis was done by prolene 8/0 sutures in an interrupted manner using high-power loupe magnification. If a marked discrepancy between the splenic and arterial graft diameters was detected, it was easily adjusted via the microsurgical methods, like oblique division of the graft artery or by leaving larger inter-suture intervals on the splenic artery side and smaller intervals on the arterial side.

Following the arterial anastomosis and ensuring its patency and adequacy, the biliary anastomosis was created using 6/0 polydioxanone, or Maxon interrupted sutures. At the end of the surgery, the viability of the spleen was assessed via gross examination and intraoperative color Doppler. Three drains were inserted, one at the right subphrenic space, one along the cut surface of the graft, and the last in the pelvis.

### Postoperative care and monitoring

Patients were initially transferred to the ICU, and then to the ward. Prophylactic antibiotics were administered, but anticoagulants were not. IV magnesium sulfate was given until oral intake was possible. Daily biochemical follow-up included complete blood count, liver and renal function tests, blood glucose, CRP, and serum amylase for the first week. Vascular patency was assessed daily via transabdominal color Doppler by an experienced hepatobiliary radiologist focusing on hepatic and portal veins, and the hepatic artery.

The surgical drains were removed when their output was below 100 ml per day and of a serous nature, neither bile nor pus. Discharge was based on the patient’s general condition and normal laboratory and radiological parameters. After discharge, regular follow-up visits were scheduled for both donors and recipients. During these visits, clinical, biochemical, and radiological assessments were done.

### Data collection and analysis

The incidence of complications (hemorrhage, thrombosis, pancreatitis) and mortality was recorded. The collected data were tabulated using the Excel software program for Windows. The quantitative data were expressed as median and range, whereas categorical data were expressed as frequency and percentage. No special statistical tests were applied in the current case series.

## Results

In this study, ten patients (1.12%) underwent SAT for hepatic arterial reconstruction out of 890 total right-lobe liver transplants performed during the same period. The age range of these patients was 41 to 56 years, with a median age of 50.5 years. The cohort included six men (60%) and four women (40%). MELD scores ranged from 2 to 48, with a median of 15, and the graft-recipient weight ratio (GRWR) ranged from 0.7 to 2.8 (median = 1.11). The primary indication for LDLT was cirrhosis in 50% of the cases (the underlying cause of cirrhosis was chronic hepatitis C), followed by hepatocellular carcinoma (HCC) in 40% on top of chronic hepatitis C cirrhosis, whereas the remaining patient had autoimmune liver disease. SAT was employed in these cases either due to marked intimal dissection of the recipient hepatic artery (7 cases – 70%) or inadequate blood flow in the same previous artery (3 cases – 30%). Previous data are summarized in Table 1.

**Table 1:** Patient demographic data and the indication of LDLT

Item	Data^1^
Age (years)	50.5 (41–56)
Gender	
-Male	6 (60%)
-Female	4 (40%)
MELD	15 (2–48)
GRWR	1.11 (0.7–2.8)
Indication of LDLT	
-Cirrhosis	5 (50%)
-HCC	4 (40%)
-Autoimmune	1 (10%)
Cause of splenic artery use	
-Hepatic artery dissection	7 (70%)
-Inadequate blood flow in the hepatic artery	3 (30%)

^1^ Median (Range); n (%)

The median operative time (total duration of the liver transplantation procedure, including all steps from incision to closure) was 797 min, ranging from 640 to 1020 min. (Table 2). Median cold ischemia duration was 28 min (range 21–36 min), while warm ischemia was 40 min (range 31–52 min). All patients received a right lobe graft. Intraoperative blood loss varied between 6 and 13 L, with a median of 10 L. Blood transfusion requirements ranged from 1 to 8 units per patient with a median of four units.

**Table 2:** Operative data of the participants

Item	Data^1^
Operative time (min)	797 (640–1020)
Cold ischemia time (min)	28 (21–36)
Warm ischemia (min	40 (31–52)
Graft type	
-Right lobe	10 (100%)
-Left lobe	0 (0%)
Blood loss (L)	10 (6–13)
Blood transfusion (unit)	4 (1–8)

^1^ Median (Range); n (%)

Table 3 Summarizes postoperative data. Postoperatively, the spleen’s gross appearance remained normal in all cases, with no instances of splenic infarction or abscess. Post-operative pancreatitis occurred in one patient (10%), with a peak serum amylase level of 2100 IU/l. three patients experienced major complications in our study. One developed intraabdominal bleeding on second postoperative day which required early reoperation and bleeding source was cut surface of the graft and was controlled with sutures and hemostatic agents. The second one developed post-operative arterial thrombosis on the third postoperative day that required thrombolysis four times. Unfortunately, this patient had an unfavorable outcome and died after 2 months from graft failure. The third developed biliary leakage and progressed to peritonitis and sepsis

**Table 3:** Follow-up data of the ten participants

Item	Data^1^
Complications	
-Biliary leakage	1 (10%)
-Pancreatitis	1 (10%)
-Splenic infarction or abscess	0 (0%)
-Intraabdominal hemorrhage	1 (10%)
-Graft arterial thrombosis	1 (10%)
Mortality	3 (30%)
Cause of mortality	
-Sepsis	2 (66.67%)
-Arterial thrombosis and graft dysfunction	1 (33.33%)

^1^ n (%)

The arterial circulation of the right lobe graft was normal in nine out of the included ten cases (90%), with a 90% patency rate. Mortality was encountered in three cases (30%), one due to arterial thrombosis and subsequent graft failure 2 months after transplantation, whereas the other two were due to sepsis. One patient developed sepsis secondary to pneumonia and died on postoperative day 21. The other one developed sepsis due to biliary leakage and peritonitis and died on postoperative day 28. At follow-up (median = 43 months), seven patients were still alive (70% survival rate).

## Discussion

Although LDLT has greatly decreased the waiting list scheduled for orthotopic liver transplantation [21, 22], each stage of the procedure itself poses a challenge for the transplant surgeon. Some difficulties, due to variations in vascular and biliary anatomy, can arise, necessitating careful surgical planning and execution.

Among these potential difficulties, hepatic artery reconstruction is a special one, especially in the setting of LDLT. Good arterial flow is crucial for the success of the transplant procedure [3]. This, in turn, needs a delicate and accurate reconstruction method that does not only depend on the surgeon’s experience but the state of the recipient’s arterial side, and the graft arterial side have their role as well.

The recipient hepatic artery may not always be feasible for direct anastomosis. It may be dissected from previous interventions like intraarterial chemoembolization [23]. Additionally, its length may be inadequate, or its blood flow may be insufficient due to stenosis or scarring from previous interventions [3, 24]. In these situations, the surgeon should seek an alternative approach [23] for arterial reconstruction to improve the postprocedural outcomes.

In the current study, operative time had a median of 797 min (640–1020). Although previous authors handling the same perspective reported that using the splenic artery for reconstruction led to a significant prolongation of operative time compared to the traditional reconstruction method (674 vs. 453 min respectively, *p* < 0.001) [3], this does not necessarily mean that the reconstruction process was extremely difficult. Of course, the transplant surgeon should initially try the traditional method of reconstruction, starting with the left or right hepatic arteries. Even if found dissected, trimming was done to a healthy part. If shortening of the artery was expected, intimal fixation was done. SAT was used as the last resort after exhausting all the earlier trials which led to more prolonged operative time, which was mainly lost on the failed traditional procedure rather than the splenic artery dissection.

Intraoperative ultrasound has become an indispensable tool in liver transplantation surgery. By providing real-time visualization of vascular structures and blood flow dynamics, it enables surgeons to assess the integrity of vascular anastomoses and optimize blood flow to the graft. This has significantly improved the safety and efficacy of LDLT by reducing the risk of post-transplant vascular complications, such as thrombosis, which can lead to graft failure and retransplantation [25].

In our surgical opinion, despite the presence of portal hypertension in most patients undergoing LDLT procedure, dissection of the splenic artery was not a great surgical problem as it was already enlarged, making it easier to identify and dissect from the surroundings. We also see that the tortuosity of the splenic artery helped in this procedure as it helped to shift it from the left abdominal side towards the right side without kinking or twisting the vascular lumen.

In our study, we did not encounter any cases of splenic infarction or abscess. However, only one patient developed pancreatitis (10%), and he was successfully managed via conservative methods.

Ligation and dissection of the splenic artery carry some risk for subsequent splenic infarction and pancreatitis, respectively, as mentioned by previous trials [26, 27]. However, preserving the short gastric vessels has been shown to keep adequate splenic vascularity, as demonstrated in angiographic studies [24].

D’Albuquerque et al. also negated the occurrence of either complication in their study which included four cases with extraanatomical hepatic artery reconstruction via the splenic artery [28]. These authors also confirmed the normal macroscopic appearance of the spleen during the surgical procedure. An additional recent study negated the incidence of any spleen-related complications after using the splenic artery. Even the patient who developed Klebsiella pneumonia was unrelated to the “post-splenectomy infection syndrome” [3].

Regarding other post-operative complications, intraabdominal hemorrhage was detected in one case (10%), while another one developed graft arterial thrombosis (10%). This patient eventually died of graft dysfunction after failed recanalization. Alim et al. reported only two major complications in the sixteen patients with SAT (12.5%). One patient had intraabdominal bleeding from the splenic artery stump that was controlled by exploration, while the other had an iatrogenic injury to the same artery during conversion of his biliary anastomosis into Roux-en-Y fashion. This case was managed by an interposition graft. Both cases did not experience mortality [3].

Another study enrolled twenty-seven cases with the traditional arterial reconstruction technique (via the hepatic arteries) and compared them to four cases that had the SAT approach. Surprisingly, the two arterial complications occurred in the traditional group. The two cases required retransplantation and died [28]. The previous findings of SAT as an arterial conduit in LDLT are safe and do not increase the risk of post-operative arterial complications.

The meta-analysis by Warsinggih et al. assessed the most suitable alternative recipient artery for anastomosis during liver transplantation when the recipient’s hepatic arteries are unusable. The study found that the splenic artery is the most favorable option for anastomosis in such cases [29].

The study by Beaurepaire et al. compared the outcomes of liver transplantation when the recipient’s hepatic artery was unusable and different alternative arteries were used for anastomosis. The four groups compared were: Splenic Artery, Celiac Trunk, Aorta, and Aorta-Prosthesis group. The study found that the splenic artery group had the best outcomes, with lower blood transfusion requirements, shorter hospital stays, and improved liver function compared to the other groups. While there was no significant difference in graft or patient survival between the groups, the splenic artery group showed superior short-term outcomes [30].

Biliary leakage was encountered in one case (10%). Uchiyama et al. reported an increased incidence of biliary complications with the extraanatomical reconstruction approaches, including SAT, as these complications were encountered in more than 50% of cases. However, another study reported that the incidence of bile leakage was statistically comparable between SAT and traditional reconstruction methods (13.5% vs. 27% respectively – *p* > 0.05) [3].

Mortality was encountered in three of our cases (30%); one was secondary to arterial thrombosis, while the other two were from sepsis. We have a 70% survival rate at the 43-month follow-up visit. In the previously mentioned study conducted by D’Albuquerque and his coworkers, the authors reported a 25.8% general mortality rate and one of these cases was from the SAT group. This patient developed pneumonia and died on the 7th postoperative day [28].

The absence of a viable retransplantation option due to limited deceased donor availability significantly impacted the outcome of patients who experienced graft failure secondary to hepatic artery thrombosis. As highlighted in our previous study, this challenge remains a significant limitation in our region [31].

Moreover, Alim et al. reported that only one patient (6.2%) had early postoperative mortality due to sepsis secondary to Klebsiella pneumonia. The other one died after 14 months due to recurrent HCC after transplantation. The one- and three-year survival rates were 93.7% and 87.5%, respectively, when SAT was done [3].

This study is limited by its small sample size and single-center scope. A comparative analysis with traditional hepatic artery reconstruction methods would have provided a more comprehensive evaluation of the SAT approach. Future research should include larger cohort and multicenter studies, with a focus on pre-and postoperative arterial Doppler indices to better assess the efficacy of different reconstruction techniques.

## Conclusion

Our study showed that employing SAT for arterial reconstruction in LDLT is a viable and safe alternative when traditional reconstruction methods are not feasible due to issues like a severe dissection of the recipient’s hepatic artery. The complication rates observed in our cohort, including instances of pancreatitis and vascular complications, align with accepted norms in complex transplant procedures. Importantly, the SAT did not significantly contribute to increased morbidity, suggesting its reliability as a secondary option.

## Data Availability

No datasets were generated or analysed during the current study.
